# Spatiotemporal characteristics and pharmacological modulation of multiple gamma oscillations in the CA1 region of the hippocampus

**DOI:** 10.3389/fncir.2014.00150

**Published:** 2015-01-12

**Authors:** Shilpashree Balakrishnan, Robert A. Pearce

**Affiliations:** ^1^Neuroscience Training Program, University of Wisconsin-MadisonMadison, WI, USA; ^2^Department of Anesthesiology, University of Wisconsin-MadisonMadison, WI, USA

**Keywords:** current source density analysis, cross frequency coupling, midazolam, atropine, theta oscillations, gamma oscillations, compartmental modeling

## Abstract

Multiple components of “γ-oscillations” between 30–170 Hz in the CA1 region of the hippocampus have been described, based on their coherence with oscillations in other brain regions and on their cross-frequency coupling with local θ-oscillations. However, it remains unclear whether the different sub-bands are generated by a single broadband oscillator coupled to multiple external inputs, or by separate oscillators that incorporate distinct circuit elements. To distinguish between these possibilities, we used high-density linear array recording electrodes in awake behaving mice to examine the spatiotemporal characteristics of γ-oscillations and their responses to midazolam and atropine. We characterized oscillations using current source density (CSD) analysis, and measured θ-γ phase-amplitude coupling by cross frequency coupling (CFC) analysis. Prominent peaks were present in the CSD signal in the mid- and distal apical dendritic layers at all frequencies, and at *stratum pyramidale* for γ_slow_ (30–45 Hz) and γ_mid_ (50–90 Hz), but not γ_fast_ (90–170 Hz) oscillations. Differences in the strength and timing of θ-γ_slow_ and θ-γ_mid_ cross frequency coupling, and a lack of coupling at the soma and mid-apical region for γ_fast_ oscillations, indicated that separate circuit components generate the three sub-bands. Midazolam altered CSD amplitudes and cross-frequency coupling in a lamina- and frequency specific manner, providing further evidence for separate generator circuits. Atropine altered CSD amplitudes and θ-γ CFC uniformly at all locations. Simulations using a detailed compartmental model were consistent with γ_slow_ and γ_mid_ oscillations driven primarily by inputs at the mid-apical dendrites, and γ_fast_ at the distal apical dendrite. Our results indicate that multiple distinct local circuits generate γ-oscillations in the CA1 region of the hippocampus, and provide detailed information about their spatiotemporal characteristics.

## Introduction

Oscillations in the brain span a wide range of frequencies and play a variety of roles in different brain structures. Different frequency oscillations are thought to be generated by distinct cellular and network mechanisms (Buzsáki, 2006). In general, higher frequency oscillations reflect the synchronous activity of locally connected cell assemblies, whereas lower frequency oscillations support longer-range coordination and communication (Singer, 1993; Singer and Gray, 1995; Fries, 2005).

In the hippocampal CA1 region, oscillations in the θ (3–12 Hz) and γ (25–170 Hz) frequency ranges are the most prominent (Vanderwolf, 1969; Leung et al., 1982; Buzsáki et al., 1983). They are thought to contribute importantly to memory formation, recall, and to other cognitive functions such as item sequencing and spatial navigation (Lisman and Idiart, 1995; Jacobs et al., 2006; Montgomery and Buzsáki, 2007; Cardin et al., 2009; Guderian et al., 2009; Tort et al., 2009; Düzel et al., 2010; Nyhus and Curran, 2010; Battaglia et al., 2011; Buzsáki and Moser, 2013). Unlike the θ-oscillation, which is sufficiently large, widespread, and regular that it creates a distinct peak in the power spectrum, γ-oscillations occur over a relatively broad range of frequencies, and there are no distinct peaks within this range. Nevertheless, based upon their coherence with γ-oscillations in other structures, γ-oscillations in the CA1 region have been separated into slow (25–50 Hz) and fast (50–140 Hz) γ-oscillations, driven by inputs from the CA3 region and entorhinal cortex (ECtx) respectively (Bragin et al., 1995; Charpak et al., 1995; Middleton et al., 2008; Colgin et al., 2009). Additional studies of cross-frequency coupling (CFC) between θ- and γ-oscillations in the CA1 region have further subdivided the faster component into mid (50–90 Hz) and high frequency oscillations (90–170 Hz) (Belluscio et al., 2012; Buzsáki and Wang, 2012; Tort et al., 2012).

Their broad frequency range, lack of distinct peaks, and local expression driven by external structures, has complicated the analysis of underlying cellular mechanisms of γ-oscillations. Locally within the CA1 region, both perisomatic inhibition and apical dendritic feed-forward inhibition are thought to contribute to the generation and expression of γ-oscillations, with periodic suppression by θ-frequency inhibitory inputs resulting in θ-γ coupling (White et al., 2000; Freund, 2003; Buzsáki and Wang, 2012; Campanac et al., 2013; Zemankovics et al., 2013; Lasztóczi and Klausberger, 2014). However, it remains unclear whether the different sub-bands are generated by a single broadband oscillator that is coupled to multiple external inputs, or by separate oscillators that incorporate distinct circuit elements.

To distinguish between these two possibilities, we examined the spatiotemporal characteristics of γ-oscillations and their responses to midazolam and atropine in awake behaving mice. Midazolam is a benzodiazepine that modulates γ subunit-containing γ-aminobutyric acid type A receptors (GABA_A_Rs). These are the major inhibitory receptors found at inhibitory synapses in the hippocampus and throughout the forebrain. (Dundee et al., 1980; Sigel and Buhr, 1997; Rudolph et al., 1999; Rudolph and Möhler, 2004). Atropine is a competitive muscarinic acetylcholine receptor (mAChR) antagonist that reduces excitability of pyramidal cells and interneurons and alters transmitter release at a subset of inhibitory nerve endings (Levey et al., 1995; Hájos et al., 1997; Qian and Saggau, 1997; Rouse et al., 2000; Cea-Del Rio et al., 2010). Atropine has been shown to alter γ-oscillations in the CA1 region *in vivo* and *in vitro* (Fellous and Sejnowski, 2000; Traub et al., 2000; Whittington et al., 2000; Mann et al., 2005). We characterized the patterns of the underlying currents using current source density (CSD) analysis, and quantified θ-γ phase-amplitude coupling using CFC analysis. We found that γ_slow_ (30–45 Hz) and γ_mid_ (50–90 Hz) oscillations had similar CSD and CFC spatial profiles under drug-free conditions, but that midazolam altered these two sub-bands differently in *stratum pyramidale*. The γ_fast_ (90–170 Hz) oscillation was distinct from both γ_slow_ and γ_mid_ oscillations in its pattern of CSD amplitudes across different laminae, as well as in its CFC profile. Atropine uniformly increased CSD amplitudes across all bands. In computer simulations employing a CA1 pyramidal neuron compartmental model, the observed physiological activity patterns were best reproduced by γ_slow_ and γ_mid_ oscillatory inputs at the level of the mid-apical dendrite and γ_fast_ oscillations at the distal apical dendrite. Our results thus indicate that distinct local circuits generate the different γ-oscillators, and that the different sub-bands are differentially modulated by midazolam but not atropine.

## Methods

### In vivo

The details of surgical methods were described previously. The results presented here belong to the same recorded data set from the same animals used for theta frequency band (4–12 Hz) oscillation analysis (Balakrishnan and Pearce, 2014). The experimental protocol was approved by the University of Wisconsin Institutional Animal Care and Use and complied with National Institutes of Health guidelines.

In brief, seven adult male homozygous mice derived from heterozygous breeding pairs carrying the GABA_A_R α5-H105R mutation were implanted with 15 μm thick, 3 mm long, 16-channel linear array recording probes (NeuroNexus Technologies “A” type probe, CM-series package, 50 μm spacing). These “pseudo wild type” animals do carry addition genetic material introduced as part of the gene-targeting strategy, and although no changes in gene expression are expected or have been described, it is possible that some exist. Surgery was performed under isoflurane anesthesia using sterile technique. The skull was secured in a stereotaxic apparatus (KOPF^®^ Instruments, Model 900) with a heated platform maintained by a circulating water bath (Stoelting^®^). Respiratory rate, temperature, and movement were monitored throughout surgery. The target recording area was the CA1 region of the dorsal hippocampus, with recording sites spanning the layers between the alveus and the hippocampal fissure. Post-surgical analgesia was provided by injecting mice with buprenorphine (0.1 mg/kg s.c.). Animals were monitored during recovery, and the first recording session was performed 7–10 days after electrode implantation.

**Figure 1:**
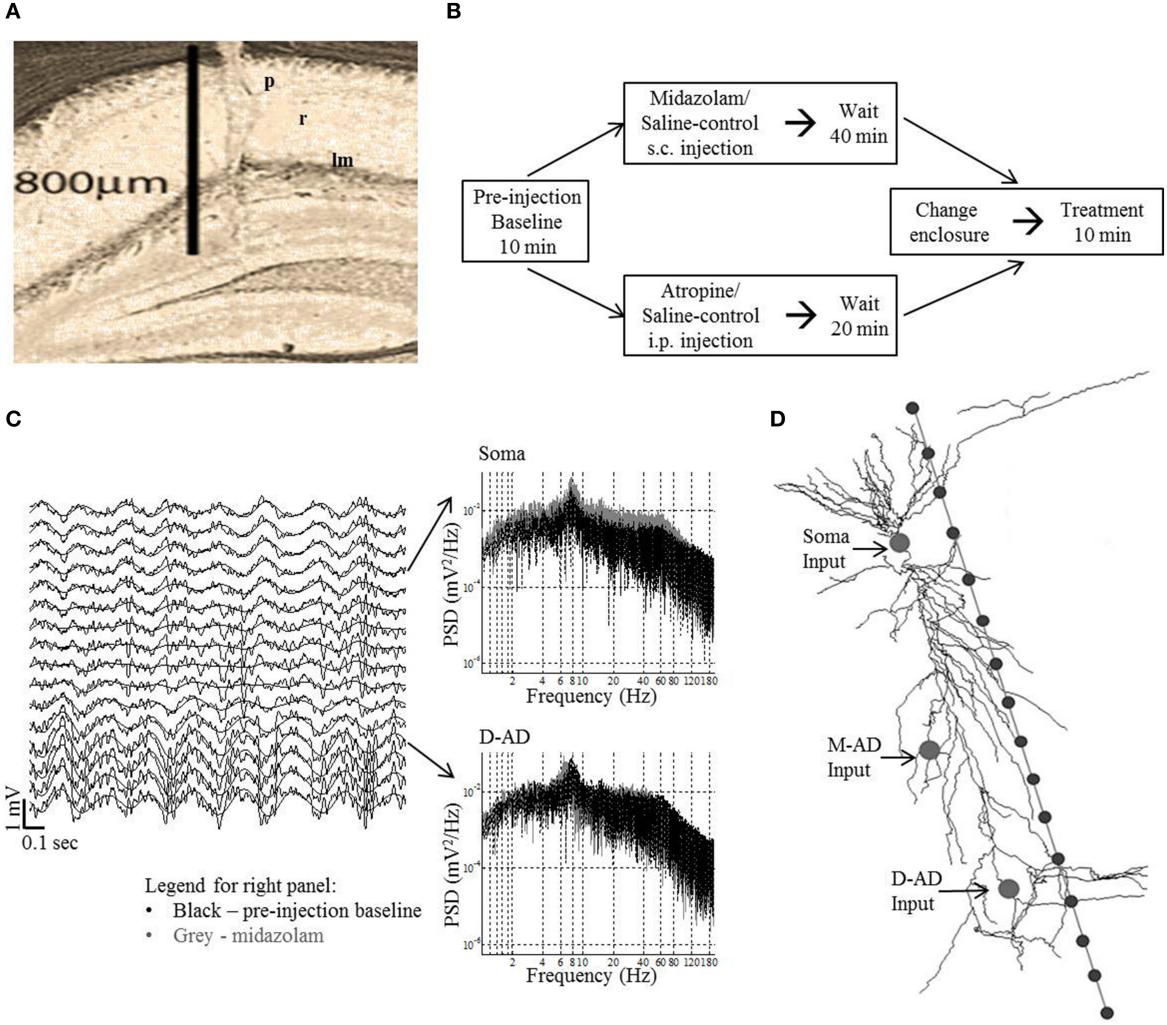
**Methods *in vivo* and *in silico*. (A)** Histology showing electrode tract in the left dorsal CA1 region: p, *stratum pyramidale*; r, *stratum radiatum*; lm, *stratum lacunosum-moleculare*. **(B)** Experimental protocol for midazolam and saline-control and atropine and saline-control administration. Data was analyzed for the first 3-min of the pre-injection baseline and treatment periods for midazolam/saline-control and for the respective 10-min period for atropine/saline-control. **(C)** Left panel—example traces (2 s) from 16 channels in an exploring mouse. The trace from recording site 4 was situated at *stratum pyramidale*. The γ-oscillations are seen to ride on the θ-oscillations (black smooth line—θ filtered signal). Right panel shows the power spectral density (PSD) at different frequencies of raw trace at soma and D-AD (black—pre-injection baseline, gray—midazolam). **(D)** Schematic of model CA1 neuron used in simulations, with 16 recording sites spaced 50 μm apart. The arrows point to the locations of the different inputs tested independently (Soma-inhibitory input alone, Mid-apical dendritic (M-AD)—excitatory/inhibitory input and distal apical dendritic (D-AD)—excitatory/inhibitory input).

Following the completion of all experiments, electrode location was verified by histological analysis (Figure 1A). In addition to estimating the laminar positions of recording sites based on histology, the location of the pyramidal cell layer was established as the recording site that showed the maximal amplitude of the ripple oscillation (170–250 Hz) during immobility.

Local field potentials (LFPs) were recorded using a Tucker Davis Technology (TDT^®^) recording system (System 3). Signals were band pass filtered from 2–6000 Hz using a zero-phase distortion digital Butterworth filter (filtfilt), acquired at a 12 KHz sampling frequency, and then extracted to a MATLAB^®^ v2008-readable format by lowpass filtering at 500 Hz and downsampling to 1 KHz. Data were analyzed using custom-written MATLAB^®^ v2012b routines. Time-stamped behavioral scoring was performed manually with a TDT^®^ scoring box (BBOX) connected to the recording system. To characterize the LFP, CSD, and drug effects on γ_slow_ (30–45 Hz), γ_mid_ (50–90 Hz), and γ_fast_ (90–170 Hz), contiguous data segments greater than 1 s, obtained during exploratory behavior, were filtered into individual frequency bands using band pass Butterworth filters (filtfilt).

Two drug treatments were used: (1) Midazolam 1.25 mg/kg subcutaneous (s.c.), which in other experiments was established as the dose that impairs freezing to context in mice by 50% (V. Rau and E.I. Eger II, unpublished data); and (2) Atropine sulfate 50 mg/kg intra-peritoneal (i.p.) which is saturating with respect to behavioral and EEG effects (Buzsáki et al., 1983; Hentschke et al., 2007). Sterile 0.9% saline was used as the vehicle/diluent and was also used for control injections.

For an individual recording session, a mouse was randomly administered drug or saline-control. Only one recording session per day was performed for each mouse. Recording sessions were comprised of three blocks (Figure 1B). (1) A mouse was placed in a rectangular glass aquarium (15 × 30 cm) with an open top, and a 10-min pre-injection block of baseline EEG/LFP activity was recorded. (2) The mouse was removed from the glass aquarium, either drug or saline was injected, and the mouse was returned to the aquarium. (3) 40 min after midazolam, or 20 min after atropine administration (or these same durations for the respective saline-controls), the mouse was moved to a transparent circular plastic enclosure of diameter 25.5 cm and a post-treatment block of EEG/LFP activity was recorded for 10 min. For midazolam, only 3-min data blocks were analyzed, as this period of time corresponded to the time during which animals explored their new environment in fear conditioning studies.

We used current source density (CSD) analysis to localize the individual gamma sub-band filtered signals and estimate their strength. The CSD was derived from band pass filtered LFP signals using the cubic spline iCSD method introduced by Pettersen et al. (2006) (MATLAB^®^ Toolbox CSDplotter). The amplitudes of the sinks/sources in the CSD signal for individual frequency bands were quantified as the root mean square of the CSD signal (rmsCSD) for each contiguous 1-s segment of filtered LFP data. Three distinct peaks were evident in the spatial distribution of the CSD. The peak observed at the somatic recording site is referred to as S_pole_, at mid-apical site as M-AD_pole_ and at the distal apical region as D-AD_pole_. For statistical evaluation, rmsCSD values for a treatment block were normalized to the median value of the pre-injection block. For each frequency band, the normalized rmsCSD values for each peak and for each drug/saline-control treatment block from all animals were pooled and plotted. To illustrate the variability between different data segments, we plotted cumulative frequency or cumulative probability distributions. One-Way ANOVA with Bonferroni posttest (GraphPad Prism^®^ v5) was used to test significance between drug and saline-control for each of the CSD poles, with each data point the average rmsCSD amplitude of 1-s data segments normalized to the median value of the pre-injection recording block.

The strength of cross frequency coupling (CFC) between the phase of the θ-oscillations and the amplitude of the γ_slow_, γ_mid_, and γ_fast_ oscillations (i.e., phase-amplitude coupling) were quantified by the modulation index (MI), as described by Tort et al. (2010). The phase-frequency was divided into 18 bins of 20°. The MI was computed in steps of 2 Hz for phase-frequency (i.e., the frequencies at which the θ-oscillation phase was used for CFC) and 2 Hz for amplitude-frequency (i.e., the frequencies at which the amplitude of the γ-oscillation for each phase-frequency was computed). Since we found that the peak frequency of θ and the maximum θ-γ coupling were both within the 6–10 Hz band, further analysis concentrated on MI values within this range. As the modulation index is a measure of deviation from uniform distribution, the values from the drug treatment blocks were directly compared to the saline-control blocks for each of the frequency bands (θ-γ_slow_/θ-γ_mid_/θ-γ_fast_) using Two-Way ANOVA with Bonferroni posttest. A minimum of 30 s of data was used to compute CFC. For the 3 min block of midazolam and its saline-control, the analyzed segments averaged 82 ± 37 and 129 ± 27 s, and for the 10 min block for atropine and its saline-control, 534 ± 51 and 270 ± 61 s respectively.

### In silico

Local field potentials recorded *in vivo* are subject to low-pass filtering by the brain tissue. Higher frequencies are attenuated more than lower frequencies, as the tissue acts like a “system of coupled RC circuits” (Bédard et al., 2006). To evaluate how this effect, together with the directional voltage attenuation seen in the CA1-PC (Carnevale et al., 1995), would affect the relationship between the input strength and the CSD profile, we simulated oscillatory inputs at three different frequencies, at three different sites corresponding to proposed physiological inputs. We then compared the spatial distribution profiles for derived CSD profiles with our *in vivo* results.

Simulations were run in the NEURON v7.1 simulation platform, using a CA1 pyramidal cell model embedded in an extracellular matrix. In brief, an “extracellular stimulation and recording” program (Carnevale, 2005) was integrated with a CA1 pyramidal cell model (Poirazi et al., 2003; Carnevale and Hines, 2009) (Senselab—ModelDB accession number 20212). We simulated oscillatory LFPs at 16 recording sites separated by 50 μm along a linear track parallel to the long-axis of the pyramidal neuron. Using an approach that we and others have followed previously (Kopell et al., 2010b; Balakrishnan and Pearce, 2014), inputs were designed as point processes, with time-varying excitatory and inhibitory synaptic inputs simulated as oscillatory conductances, the reversal potentials of which were set to 0 mV or −75 mV respectively (Figure 1D). Values for conductances were chosen such that they were subthreshold for action potential generation (somatic inhibitory conductance = 0.003 mho/cm^2^; dendritic excitatory conductance = 0.0004 mho/cm^2^; dendritic inhibitory conductance = 0.01 mho/cm^2^. Three input locations were selected for independent simulations: (1) Soma input (inhibitory only; compartment = soma[1]); (2) Mid-apical dendritic input (inhibitory or excitatory; compartment = apical_dendrite[68]); and (3) Distal apical dendritic input (inhibitory or excitatory; compartment = apical_dendrite[92]) (Figure 1D). Three frequencies of sinusoidally varying conductances—30 Hz (~γ_slow_), 59 Hz (~γ_mid_) and 111 Hz (~γ_fast_)—were independently simulated at each of the locations. Except for these characteristics of oscillatory inputs, model parameters were the same as those we used previously for our simulations of θ-band oscillations (Balakrishnan and Pearce, 2014). CSD profiles were derived from the 16-channel LFPs, and quantified using root mean square over the last 1750 ms of the 2000 ms simulation (the first 250 ms of data discarded to eliminate fluctuations due to model settling time). The CSD profiles were computed in same fashion as for *in vivo* data.

## Results

### CSD spatial profiles

**Figure 2:**
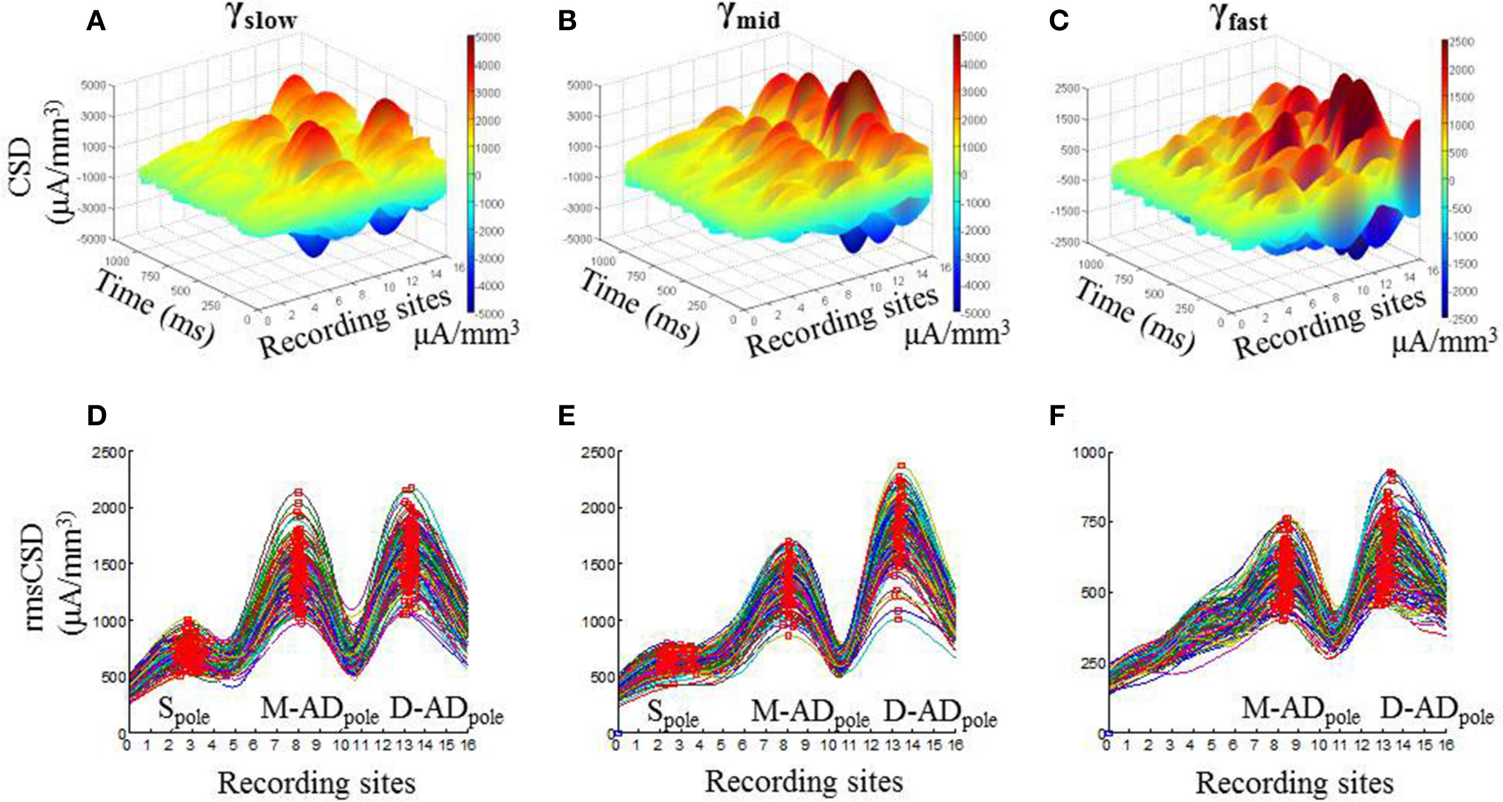
**Spatial profile of CSD derived from LFP for gamma oscillations in CA1**. One second example 3-D CSD plots of **(A)** γ_slow_ (30–45 Hz), **(B)** γ_mid_ (50–90 Hz), and **(C)** γ_fast_ (90–170 Hz) oscillations. (**D–F)** Each trace shows root mean squared values of CSD (rmsCSD) for 1-s data segments during the pre-injection baseline recording session in an exploring mouse, for γ_slow_, γ_mid_, and γ_fast_ oscillations respectively. In all bands the amplitude of the CSD was highest at the fissure (electrodes 13–14), with a large peak also present in the mid-apical region. The relative amplitude of the somatic peak decreased as the frequency increased, and was absent for γ_fast_.

*In vivo* recordings of LFPs obtained during open field exploration showed that higher frequency γ-oscillations were present together with lower frequency θ-oscillations (Figure 1C, Supplementary Figure 1), as other investigators have reported previously (Tort et al., 2008; Colgin et al., 2009; Belluscio et al., 2012). We examined the spatial pattern of the current sinks and sources that produce each of the three higher frequency bands using CSD analysis. Figures 2A–C show examples from an individual animal of the CSD signal during 1 s of continuous exploration, plotted as a function of position and time in a 3-dimensional representation, for γ_slow_ (30–45 Hz—Figure 2A), γ_mid_ (50–90 Hz—Figure 2B) and γ_fast_ (90–170 Hz—Figure 2C) activity. Distinct peaks, corresponding to individual poles of oscillating dipoles, were especially evident at sites positioned within the mid-apical dendrite (M-AD, recording sites 7–9) and distal apical dendrite layers (D-AD, recording sites 12–15), but also near the soma (S, recording sites 2–3) in this example.

To visualize the pattern and variation in the CSD poles over the full analysis period, we calculated the root mean square of the CSD signal (rmsCSD) for each 1 s data segment, and plotted the combined data for each of the frequency bands (Figures 2D–F). Again, it was evident that the D-AD_pole_ and M-AD_pole_ amplitudes were substantially larger than the S_pole_ at all frequencies. In addition, compared to the D-AD_pole_ and M-AD_pole_, which were consistently present in all recordings in all animals, the S_pole_ showed substantial variability between animals; it was not discernible in the γ_slow_ band in two mice, or in the γ_mid_ band in four mice, and it was completely absent in the γ_fast_ oscillations in all mice.

This analysis thus showed that γ-oscillations are driven by current sources and sinks concentrated in three distinct regions—the soma, the mid-apical dendrite, and the distal apical dendrite—but that the two dendritic inputs predominate for all three sub-bands.

### Effect of midazolam on rmsCSD amplitudes

**Figure 3:**
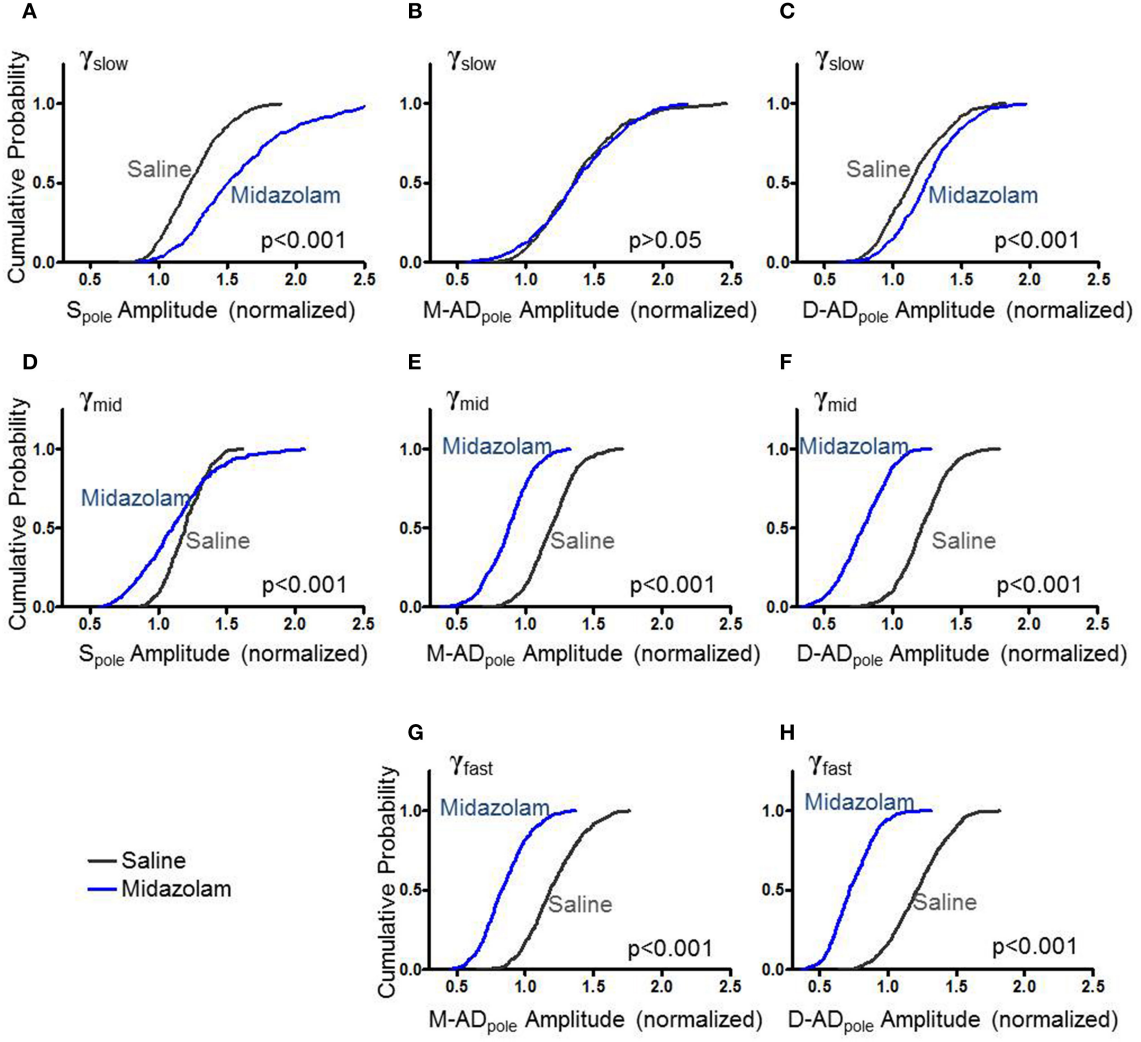
**Effect of midazolam on the amplitude of rmsCSD peaks of γ_slow_ (A–C), γ_mid_ (D–F), and γ_fast_ oscillations (G,H). (A)** Midazolam significantly increased the rmsCSD amplitude of γ_slow_ oscillations at the somatic region (S_pole_) compared to saline-control. **(B)** Midazolam did not change the amplitude of γ_slow_ at the mid-apical dendritic peak (M-AD_pole_). **(C)** There was a slight but significant increase in the amplitude of γ_slow_ at the distal apical dendrite rmsCSD peak (D-AD_pole_). **(D–F)** Midazolam decreased the rmsCSD amplitudes of γ_mid_ oscillations at all locations. **(G,H)** Midazolam decreased the rmsCSD amplitudes of M-AD_pole_ and D-AD_pole_ of γ_fast_ oscillations; no S_pole_ was present for this component.

Previous studies have implicated GABAergic synaptic transmission in the generation of γ-oscillations expressed by hippocampal slices (Traub et al., 2000, 2003). To test the effect of the GABA_A_ receptor modulator midazolam on γ-oscillations *in vivo*, we administered a sub-hypnotic but amnestic dose and assessed effects on the different sub-bands by comparing the cumulative probability distributions of rmsCSD peaks for γ_slow_, γ_mid_ and γ_fast_ oscillations (Figure 3). For γ_slow_, midazolam increased the amplitude of the S_pole_ (Figure 3A: midazolam/saline-control, *n* = 402/367 data points from 5/4 mice *p* < 0.001 One-Way ANOVA) and the D-AD_pole_ (Figure 3C: midazolam/saline-control, *n* = 458/503 data points from 7/6 mice *p* < 0.001 One-Way ANOVA), but had no effect on the M-AD_pole_ (Figure 3B: midazolam/saline-control, *n* = 458/503 data points from 7/6 mice). In contrast, for γ_mid_ and γ_fast_ oscillations, midazolam significantly decreased the amplitude of rmsCSD at all locations (γ_mid_—Figures 3D–F: midazolam/saline-control, S_pole_: *n* = 266/234 data points from 3/3 mice *p* < 0.001; M-AD and D-AD_pole_: *n* = 508/595 data points from 7/6 mice *p* < 0.001, γ_fast_—Figures 3G,H: midazolam/saline-control, *n* = 535(M-AD_pole_), 531(D-AD_pole_)/648(M-AD_pole_), 647(D-AD_pole_) data points from 7/6 mice *p* < 0.001 One-Way ANOVA).

This pattern of effects—an increase in the slowest component primarily at the soma, and decreases in the faster two components at all locations—is consistent with the participation of GABAergic inhibition in the generation or expression of all three sub-bands. The location- and frequency-dependent pattern of changes suggests that oscillations in the different frequency bands arise through distinct cellular mechanisms, with midazolam differentially modulating their strength.

### Atropine increased rmsCSD amplitudes for all frequency bands at all locations

**Figure 4:**
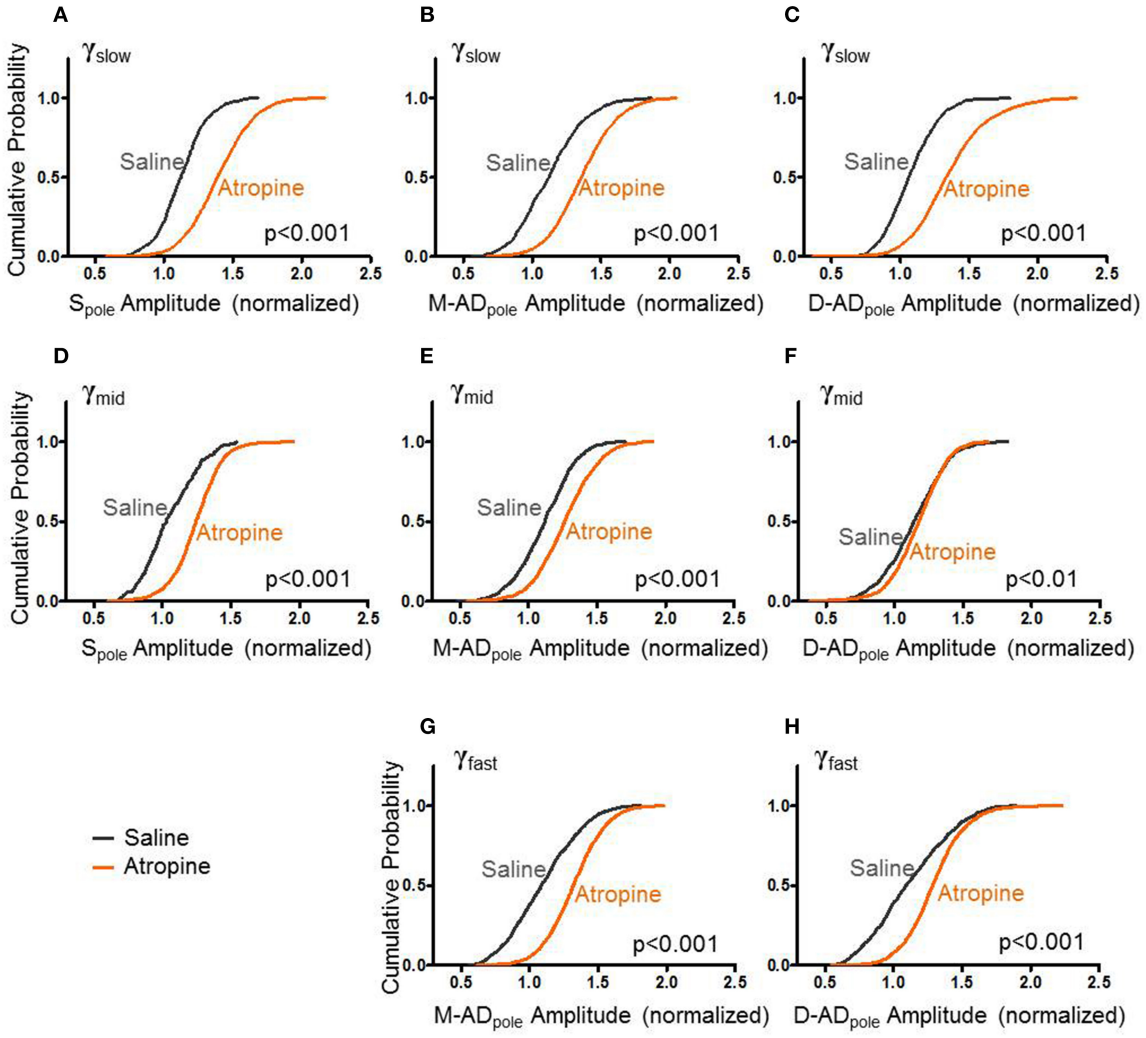
**Atropine increases amplitude of CSD for all bands at all sites. (A–C)** With respect to saline-control, atropine significantly increased the amplitudes of S_pole_, M-AD_pole_, and D-AD_pole_ respectively of γ_slow_ oscillations for all animals (atropine/saline-control, S_pole_: *n* = 1875/871 data points from 3/3 mice; M-AD and D-AD_pole_: *n* = 2154(M-AD_pole_), 2192(D-AD_pole_)/871 data points from 5/4 mice *p* < 0.001 One-Way ANOVA). **(D–F)**, Similarly for γ_mid_, atropine significantly increased the rmsCSD amplitudes at all locations, though the difference was less at the D-AD_pole_ (atropine/saline-control, S_pole_: *n* = 991/175 data points from 3/1 mice and M-AD_pole_: *n* = 1755/1054 data points from 5/4 mice *p* < 0.001; D-AD_pole_ *n* = 1755/1054 data points from 5/4 mice *p* = 0.001–0.01) One-Way ANOVA. **(G,H)** For γ_fast_ oscillations, atropine significantly increased the amplitude of rmsCSD at the M-AD_pole_ and D-AD_pole_ (atropine/saline-control, *n* = 2671(M-AD_pole_), 2670(D-AD_pole_)/1205 data points from 5/4 mice *p* < 0.001 One-Way ANOVA).

Like midazolam, atropine impairs hippocampal function. However, it does so through a distinct molecular mechanism—by antagonizing mAChRs. To further explore the pharmacologic sensitivities of the different sub-bands, we administered atropine and measured its effect γ_slow_, γ_mid_, and γ_fast_ oscillations. Figure 4 summarizes the effects of atropine (orange) compared to saline-controls (gray) on the rmsCSD peaks of γ_slow_, γ_mid_, and γ_fast_ oscillation activity in the CA1 region. Unlike midazolam, which had different effects on different peaks, atropine increased the amplitudes of γ_slow_, γ_mid_, and γ_fast_ oscillations at all locations.

### Cross-frequency coupling profiles

**Figure 5:**
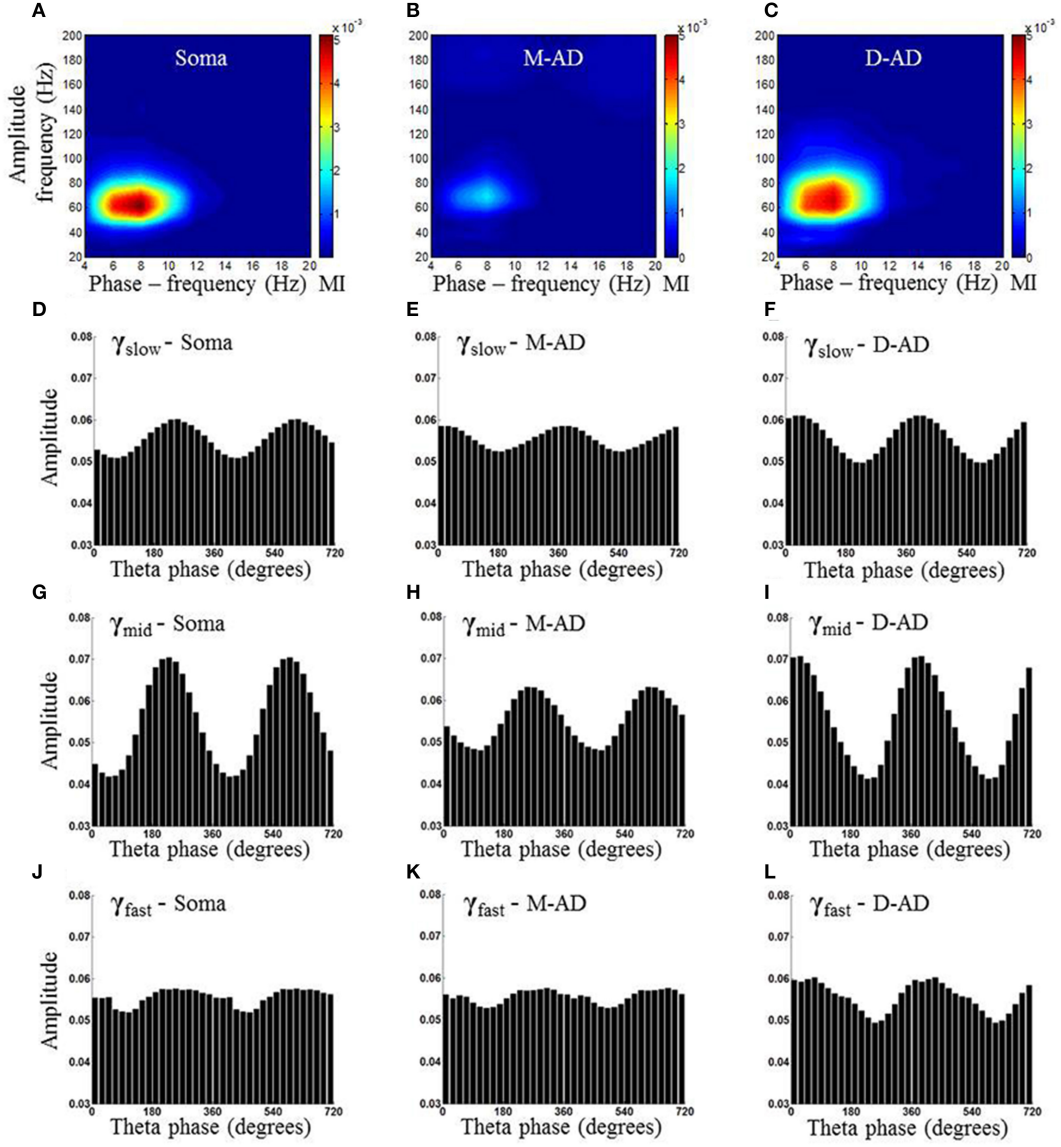
**Example of comodulogram and phase amplitude coupling at somatic, mid-apical and distal apical dendrite recording sites, for a single animal. (A–C)** Comodulogram showing the modulation Index (MI) plotted as a function of phase-frequency and amplitude-frequency from a single animal. Hotter colors indicate larger amplitude modulation. **(D,G,J)** γ_slow_ (30–45 Hz)/γ_mid_(50–90 Hz) /γ_fast_ (90–170 Hz) amplitude modulation by θ (6–10 Hz) phase, binned into 18 subdivisions of 20° each at the somatic recording site. (**E,H,K)** The γ_slow_, γ_mid_, and γ_fast_ amplitudes from the recording site at the mid-apical dendrite (M-AD) shows relative amplitude modulation by θ similar to that seen at the somatic site; however the phase of θ at which gamma amplitude was maximum were offset by 120° for γ_slow_ and 20° for γ_mid_. **(F,I,L)** At the distal apical dendrite (D-AD) the amplitude coupling was anti-phasic (offset by 180°) for γ_slow_ and γ_mid_ with respect to soma. In addition the θ-γ_fast_ coupling was visible primarily only at D-AD [Phase amplitude coupling measurements shown in this figure were obtained from pre-injection baseline data in an exploring animal using the method described by Tort et al. (2010)].

Previous studies have found that the different sub-bands differ in cross-frequency coupling with the θ-oscillation (Tort et al., 2008, 2012; Colgin et al., 2009; Belluscio et al., 2012; Scheffer-Teixeira et al., 2012). We explored layer-specific characteristics of CFC by measuring the amplitude of γ-oscillations as a function of the phase of the local θ-oscillation. Figure 5 shows an example from one animal during the baseline-recording period, prior to injecting a drug or saline. Here, the amplitude of the γ-oscillation is plotted as a function of the phase of the θ-oscillation, for all three sub-bands at each of three recording sites (Figures 5A,D,G,J: recording site at soma, Figures 5B,E,H,K: M-AD, Figures 5C,F,I,L: D-AD), where the top panel (Figures 5A–C) shows the comodulogram of the modulation index (MI) that quantifies the modulation of gamma at each of the sites. It is apparent that modulation was greater for γ_mid_ than for γ_slow_ or γ_fast_ oscillations at all locations. This pattern was observed consistently across animals; on average, the amplitude modulation of γ_mid_ was ~6 times that of γ_slow_ and ~4 times that of the γ_fast_ oscillation, as assessed by comparing the modulation index (MI) for each of the sub-bands at D-AD. Although the rmsCSD amplitudes of γ_slow_ and γ_mid_ were higher in the M-AD than at the soma (Figures 2D,E), the phase-amplitude coupling was lowest in the M-AD, confirming that MI values are independent of CSD amplitude (Tort et al., 2010).

A second difference between the CFC of the three sub-bands was evident in the timing of their phase-amplitude coupling with the local θ-oscillation. In all cases, the phase at which amplitude modulation was the greatest was shifted by 180° across layers, with the peak at ~240° at the soma coinciding with a trough at the D-AD (Figures 5D,G,J,F,I,L). This pattern matches the 180° phase reversal of the θ-filtered LFP in the CA1 region (Green et al., 1960; Winson, 1974; Bland et al., 1975; Balakrishnan and Pearce, 2014). However, the timing of the CFC in the mid-apical region differed: for γ_mid_ the phase of maximum amplitude modulation matched that of the soma, whereas the phase of maximum amplitude modulation of γ_slow_ matched the timing of the D-AD.

Taken together, our findings that the different sub-bands differed in the strength of their cross frequency coupling, and in the timing of the coupling in the mid-apical region, support a model in which separate oscillatory circuits generate γ_slow_, γ_mid_, and γ_fast_ oscillations; a single broadband oscillator would be expected to be more uniformly coupled in strength and timing to the phase of the modulating θ-oscillation.

### Effect of midazolam and atropine on CFC

**Figure 6:**
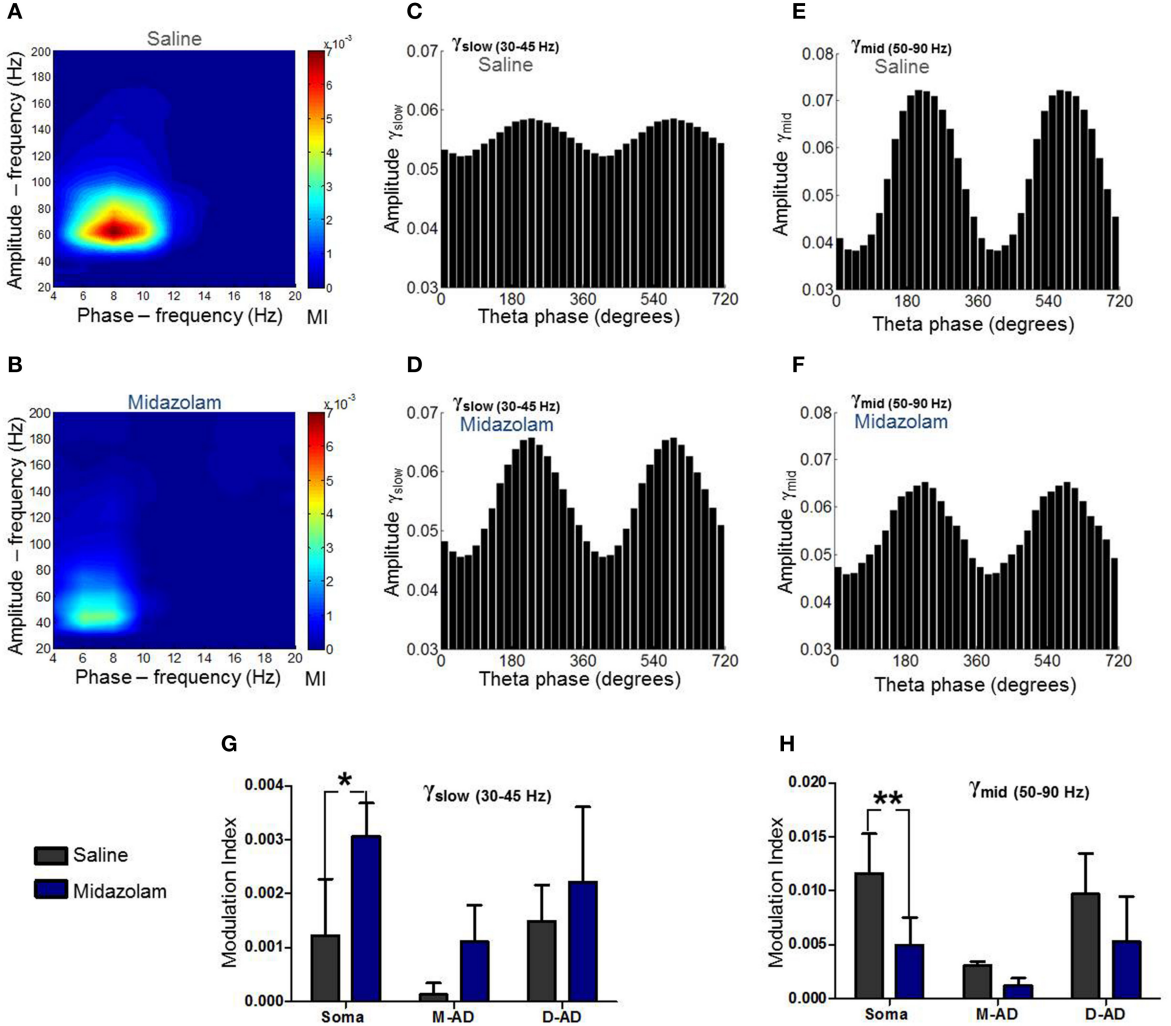
**Summary of midazolam effects on θ-γ_slow_ and θ-γ_mid_ cross frequency coupling. (A)** Comodulogram at the somatic recording site following saline administration. **(B)** Comodulogram following midazolam administration at the somatic recording site, from the same mouse shown in (**A**). Peak MI values were shifted to lower amplitude- and phase-frequencies. **(C,D)** γ_slow_ amplitude modulation at the somatic recording site as a function of θ-phase following administration of saline **(C)** or midazolam **(D)**. **(E,F)** γ_mid_ amplitude modulation at the somatic recording site as a function of θ-phase following administration of saline **(E)** or midazolam **(F)**. **(G,H)** Grouped data from all animals. Midazolam increased θ-γ_slow_ coupling **(G)** and decreased θ-γ_mid_ coupling **(H)** at the somatic recording site. No significant effects were seen at mid-apical dendrite (M-AD) or distal-apical dendrites (D-AD). ^*^p < 0.05; ^**^p < 0.01.

**Figure 7:**
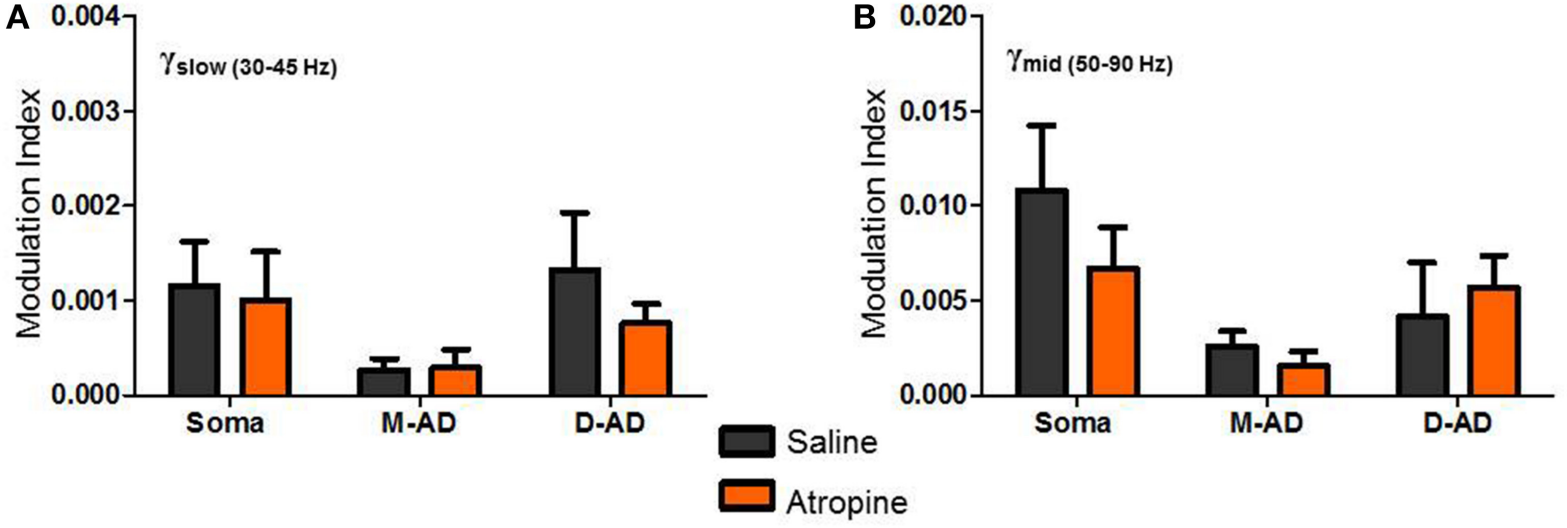
**Atropine effect on θ-γ_slow_ and θ-γ_mid_ cross frequency coupling. (A,B)** No significant changes in Modulation Index (MI) were observed following atropine administration compared to saline-control, for θ-γ_slow_ **(A)** or θ-γ_mid_ cross frequency coupling, at the somatic, mid-apical dendrite (M-AD) or distal apical dendritic (D-AD) recording sites.

In addition to characterizing their CFCs under drug-free conditions, we examined the effects of midazolam and atropine on the three sub-bands. An example of the effect of midazolam at the somatic recording site in an individual animal is shown in Figures 6A–F, comparing oscillations following administration of midazolam (Figures 6B,D,F) vs. saline (Figures 6A,C,E). Average effects on MI in all animals are shown in Figures 6G,H for midazolam and its saline-control, and Figure 7 for atropine and its saline-control. Following administration of midazolam, both the peak frequency of the modulating θ-oscillation and the peak frequency of the modulated γ-oscillation shifted to lower values (Figures 6A,B), so that the maximum modulation occurred within the γ_slow_ frequency band rather than γ_mid_, as seen during the pre-injection baseline (Figure 5) and following saline injection (Figures 6A,C,E).

Comparing the modulation index values for all animals at the somatic recording site, we found a significant increase in the MI for θ-γ_slow_ CFC (Figure 6G: midazolam/saline-control, *n* = 5/5 *p* < 0.01, Two-Way ANOVA) but no significant change at the M-AD and D-AD recording sites (Figure 6G: midazolam/saline-control, M-AD: *n* = 3/5, D-AD: *n* = 6/6 *p* > 0.05, Two-Way ANOVA). In contrast, midazolam significantly decreased the MI of θ-γ_mid_ CFC at the somatic recording site (Figure 6H: midazolam/saline-control, *n* = 5/5 *p* < 0.01, Two-Way ANOVA) and again had no significant effects at the M-AD and D-AD recording sites (Figure 6H: midazolam/saline-control, M-AD: *n* = 3/5, D-AD: *n* = 6/6 *p* > 0.05, Two-Way ANOVA). Atropine had no significant effects on the CFCs for either θ-γ_slow_ (Figure 7A) or θ-γ_mid_ (Figure 7B) at any of the recording sites (Figures 7A,B: atropine/saline-control, *n* = 4/5 *p* > 0.05, Two-Way ANOVA). For the θ-γ_fast_ CFC, only modulation of the D-AD was large enough to analyze, and there were no effects on MI for either drug (midazolam/saline-control, *n* = 6/6 *p* > 0.05, Two-Way ANOVA; atropine/saline-control, *n* = 4/5 *p* > 0.05, Two-Way ANOVA) (data not shown). Neither midazolam nor atropine altered the phase of θ at which the amplitude peaks appeared for any of the sub-bands.

The lack of effect of atropine on CFC indicates that although muscarinic receptors modulate the circuits that generate γ-oscillations, they do not modulate the components that underlie phase-amplitude coupling within the CA1 network. By contrast, the opposite effects of midazolam on θ-γ_slow_ and θ–γ_mid_ CFC is consistent with coupling between inhibitory circuits as the mechanism for θ-γ coupling, and it provides further evidence that the underlying circuitry differs for these two oscillations.

### Compartmental modeling

**Figure 8:**
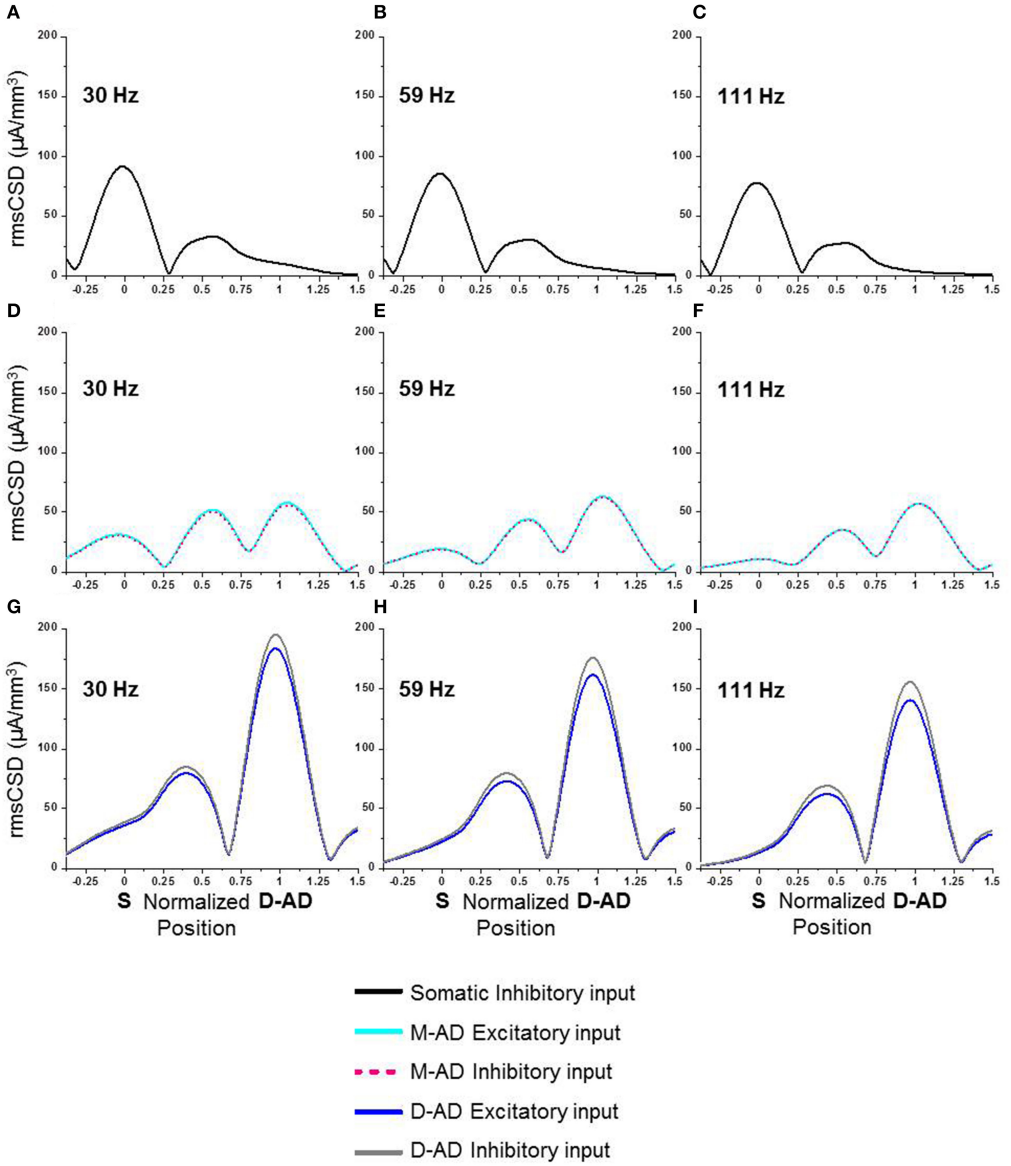
**Computer simulations of spatial spread of γ-oscillations at different frequencies in a compartment model of a CA1 pyramidal neuron. (A–C)** Inhibitory input imposed exclusively at the soma. **(D–F)** Excitatory (cyan) or inhibitory (pink) input at the mid-apical dendrite (M-AD). **(G–I)** Excitatory (blue) or inhibitory (gray) input at the distal apical dendrite (D-AD). **(A,D,G)** 30 Hz input (~γ_slow_) at soma and distal dendrite spread to mid-apical dendrite, and M-AD input spread to both soma and D-AD. **(B,E,H)** 59 Hz input (~γ_mid_) produced a pattern similar to 30 Hz input, but with less spread to soma and more to D-AD. **(C,F,I)** 111 Hz input (~γ_fast_) produced a pattern similar to 30 and 59 Hz inputs, but with even greater attenuation of spread to the soma. X-axis positions are normalized, with “0” corresponding to soma and “1” to the D-AD inputs.

The *in vivo* rmsCSD spatial profiles showed that the M-AD_pole_ and D-AD_pole_ were larger than the D-AD_pole_, for all three sub-bands (Figure 2). These CSD peaks could be produced by local active synaptic inputs, or they could reflect the locations of passive returns from spatially distant inputs, as occurs for θ-frequency oscillations (Balakrishnan and Pearce, 2014). We used computer simulations to determine how single inputs at different frequencies (30, 59, 111 Hz) and locations (soma, mid-apical, distal-apical dendrite) would be expected to influence the rmsCSD profiles in the presence of frequency-dependent attenuation (Figure 8). Excitatory and inhibitory inputs were simulated at each location as oscillatory conductance changes. The CSD was then derived from the extracellular LFP, and its spatial spread and amplitude pattern were evaluated. The patterns produced by oscillatory mid-apical dendrite inputs at 30 and 59 Hz matched the CSD spatial profiles that we observed *in vivo* for γ_slow_ and γ_mid_ (Figures 8D,E), with large amplitude oscillations present at both M-AD and D-AD sites and a smaller amplitude oscillation at the soma. The pattern produced by an oscillatory input at the D-AD matched the *in vivo* profile of the γ_fast_ oscillation, which lacked a peak at the soma (c.f. Figures 2, 8). However, simulating an oscillatory input at the soma produced a large-amplitude oscillation at the soma, a smaller peak in the M-AD region, and no peak at the D-AD—a pattern that did not match any profile recorded *in vivo*.

Since changes in membrane shunting have been invoked for many drug effects, including midazolam, we also considered how a midazolam-induced increase in membrane conductance might influence local CSD amplitude. Contrary to our physiological results, an increase in membrane leak conductance led to a decrease in CSD amplitudes at the soma, both for oblique excitatory as well as somatic inhibitory input (data not shown). Therefore, we conclude that midazolam effects cannot be simply a change in passive propagation due to membrane shunting effects.

These modeling results thus showed that the spatiotemporal characteristics we observed for γ_slow_ and γ_mid_ oscillations matched the expected patterns produced by M-AD inputs, and that the profile for γ_fast_ oscillations matched the D-AD input pattern. We expect that combinations of oscillatory drivers would generate profiles with mixed characteristics, but we did not explore CSD profiles produced by multiple inputs in any detail.

## Discussion

In this study we sought evidence for local differences in the expression or generation of γ_slow_ (30–45 Hz), γ_mid_ (50–90 Hz), and γ_fast_ (90–170 Hz) oscillations in the CA1 region of the hippocampus, based on their spatiotemporal profiles, their cross frequency coupling with the local θ-oscillation, and their pharmacological modulation. CSD analysis revealed prominent peaks in the mid- and distal-apical dendrites for all three sub-bands, and a smaller peak at the soma that was variably present for the γ_slow_ and γ_mid_, but absent for γ_fast_ oscillations. Differences in the strength and timing of θ-γ_slow_ and θ-γ_mid_ cross frequency coupling, and a lack of coupling at the soma and mid-apical region for γ_fast_ oscillations, suggested that separate biophysical processes generate the three sub-bands. The lamina- and frequency specific modulation by midazolam of both CSD amplitude and cross-frequency coupling provided further evidence for separate underlying generator circuits. Based on these results, we conclude that distinct local circuits generate γ_slow_, γ_mid_, and γ_fast_ oscillations in the CA1 region of the hippocampus.

### Multiple γ-oscillations in the hippocampus

Investigations conducted in recent years by a number of laboratories have revealed that “γ-oscillations” can be divided into several distinct components. As new information has emerged, the nomenclature and definition of gamma sub-bands has varied. Based upon their coherence and phase locking with cells in other brain regions, “slow” (25–50 Hz) and “fast” (50–150 Hz) γ-oscillations in the CA1 region were described initially, with “slow gamma” driven or entrained by input from the CA3 region, and “fast gamma” by input from the ECtx (Bragin et al., 1995; Charpak et al., 1995; Middleton et al., 2008; Colgin et al., 2009). Detailed characterization of θ-γ cross frequency coupling revealed distinct “high frequency oscillations” (HFOs—also referred to as ε-oscillations) in the 110–160 Hz range, and indicated that γ-oscillations in the lower frequency range can be separated into “γ-slow” (30–60 Hz) and “γ-mid” (60–100 Hz) oscillations (Scheffer-Teixeira et al., 2012; Tort et al., 2012). Studies of θ-γ phase-phase coupling, another form of CFC, revealed additional frequency-dependent differences (Belluscio et al., 2012). Our present results further support the separation of “γ-oscillations” into three distinct sub-bands generated by different biophysical processes. In keeping with the nomenclature of Belluscio et al. (2012), we refer to them here as γ_slow_, γ_mid_, and γ_fast_ oscillations.

### Mechanism of γ-oscillation generation—contribution of external inputs

Oscillations in the CA1 region at frequencies below ~70 Hz were proposed to be entrained or driven by γ-oscillations in the CA3 region (Middleton et al., 2008; Colgin et al., 2009). Our present results support this hypothesis, as patterns of CSD spatial profiles derived from physiological recordings *in vivo* (Figure 2) matched computer simulations with inputs in the mid-apical region (Figures 8D–F). Thus, active inputs in the mid-apical region appear to contribute to γ_slow_ and γ_mid_ oscillations. Interestingly, this situation is unlike that for θ-oscillations, in which case a passive mid-apical peak is created by the overlapping return currents from active inputs located at the soma and distal apical dendrites (Balakrishnan and Pearce, 2014).

By contrast, the maximal current sources and sinks driving γ_fast_ oscillations were located in *stratum lacunosum-moleculare*. θ-γ_fast_ CFC was also maximal in this layer. In keeping with prior suggestions that input from the ECtx drives or entrains γ-oscillations at higher frequencies, we found a correspondence between physiological results showing that the S_pole_ is absent *in vivo* for γ_fast_ (Figure 2F), as it is in computer simulations with distal apical dendritic input (Figures 8G–I). However, in some prior studies, γ_fast_ oscillations were reported to be concentrated in the superficial layers, i.e., *stratum oriens* and *stratum pyramidale* (Belluscio et al., 2012; Scheffer-Teixeira et al., 2012; Tort et al., 2012). Our present finding that γ_fast_ oscillations are maximal in *stratum lacunosum-moleculare*, in register with afferent input from ECtx, thus supports the association between γ_fast_ in CA1 and ECtx (Lasztóczi and Klausberger, 2014).

### Modulation of γ-oscillations by midazolam

Since we used an amplitude-independent measure of cross frequency coupling (Tort et al., 2010), the differences between sub-bands in CFC characteristics and drug responses (Figures 5, 6G,H) provided additional evidence of different intrinsic and network mechanisms. Were there a single broad-band oscillator that was simply shifted to lower frequencies by midazolam, power might have appeared to go down in the higher frequency band, and up in the lower frequency band, but these changes would not have altered the characteristics of CFCs within those bands. We found the most prominent differences in *stratum pyramidale*, where midazolam increased γ_slow_ CSD amplitude and θ-γ_slow_ CFC, but decreased γ_mid_ CSD amplitude and θ-γ_mid_ CFC (Figures 3A,D, 6). The γ_mid_ and γ_fast_ oscillations responded similarly to midazolam as well as atropine; however their baseline CSD and CFC characteristics differed, as described above.

One of the models proposed for generation of gamma oscillations involves an interaction between excitatory neurons and inhibitory interneurons (Whittington et al., 2000; Kopell et al., 2010a). Previous research has shown that PV-BCs resonate at γ-frequency (Pike et al., 2000) and impose a strong inhibition at the soma of CA1-PCs (Freund and Buzsáki, 1996; Buzsáki, 2002). Also, PV-BCs fire at ~35 Hz (Campanac et al., 2013), and PV-BCs regulate perisomatic γ-oscillations without any effect on γ-oscillations in dendritic regions (Lasztóczi and Klausberger, 2014). Taken together, these findings suggest that the contribution of PV-BCs is restricted to the γ_slow_ frequency band. These BC's in turn are known to receive inhibitory input from O-LM interneurons (Elfant et al., 2008). Hence the effects of midazolam on the CSD amplitude of γ_slow_, and on θ-γ_slow_ CFC at *stratum pyramidale*, likely arise from its modulation of inhibitory synapses embedded in a network involving interactions between CA1-PCs, basket cells, and O-LM interneurons, and not exclusively on CA1-PC GABA_A_Rs. Moreover, the differential distribution of GABA_A_Rs composed of different subunits on CA1-PCs and interneurons likely influences midazolam's effects as well (Nusser et al., 1996; Somogyi et al., 1996; Brünig et al., 2002; Serwanski et al., 2006; Salesse et al., 2011).

In contrast to γ_slow_, midazolam decreased the CSD amplitude of γ_mid_ at all locations, and decreased the θ-γ_mid_ CFC at the soma. Since midazolam enhances GABAergic inhibition (Sigel and Buhr, 1997; Rudolph et al., 1999; Rudolph and Möhler, 2004), and inhibition is thought to contribute critically to the generation of γ-oscillations (Traub et al., 2000, 2003; Jackson et al., 2011; Chen et al., 2014), this finding is therefore somewhat counterintuitive. We suggest two possible explanations. First, γ_mid_ might be generated by temporally and spatially overlapping excitatory and inhibitory dendritic inputs. In this case, enhanced inhibition may offset excitation and thereby reduce oscillation amplitude, as suggested for θ-oscillations based on computer simulations (Balakrishnan and Pearce, 2014). Indeed, a recent study described interneurons in the *stratum lacunosum moleculare* coupled to 60–100 Hz oscillations of urethane-anesthetized rats, and slightly higher frequencies in awake head-fixed mice, recorded exclusively from the apical dendrite region (Lasztóczi and Klausberger, 2014). Midazolam could increase the strength of GABAergic input onto pyramidal cells from such interneurons, offsetting excitatory components of γ_mid_ and/or γ_fast_ oscillations in the CA1 region. Alternatively, midazolam may globally suppress oscillations in the inhibitory networks that generate γ_mid_ and γ_fast_ oscillations, so that even if inhibitory input onto pyramidal neurons is enhanced, the net effect is a reduced inhibitory current in CA1-PCs. It is also possible that midazolam differentially affects the CA1 local circuitry by prolonging the GABA IPSC decay constant such that it is not able to resonate at the higher frequency inputs. Our present results do not allow us to distinguish between these possible explanations.

The midazolam-induced decrease in θ-γ_mid_ CFC matches the change produced by deletion of the γ2 subunit in fast-spiking interneurons (Wulff et al., 2009), though the exact nature of the interneuronal and CA1-PC connections that participate in the θ-γ_mid_ circuit modulated by midazolam remain undefined. Unlike its effect on CFC at the somatic recording site, midazolam did not significantly alter the CFC at the distal apical dendrite for either γ_slow_ or γ_mid_. This finding provides a further indication that the different sub-bands are produced by distinct biophysical mechanisms.

Since inhibitory circuitry is thought to contribute to generation of oscillations, and oscillations are important for memory function, it is curious that at least some types of oscillations are enhanced by midazolam, a drug that disrupts memory. How can these observations be reconciled? The answer may lie in the need for precise timing. Phase-amplitude coupling, which is the most widely recognized and studied form of CFC, is thought to arise by the interaction of slow spiking interneurons (e.g., O-LM interneurons) with fast spiking interneurons (e.g., basket cells) (White et al., 2000; Rotstein et al., 2005; Tort et al., 2007). This coupling was proposed to be a mechanism by which information processing and encoding occurs in neural assemblies (Lisman and Idiart, 1995; Lakatos et al., 2005; Senior et al., 2008), and to be important for memory in humans (Axmacher et al., 2010), as it predicts learning (Tort et al., 2009) and overall memory performance (Shirvalkar et al., 2010; Friese et al., 2013). We found that when midazolam was administered at the ED_50_ dose for amnesia in mice, there were changes in CFC of θ-γ_slow_ and θ-γ_mid_ at *stratum pyramidale*. These results indicate that midazolam and other drugs that produce a state of “conscious amnesia” might impair memory by disrupting essential timing mechanisms rather than suppressing overall circuit activity.

### Modulation of γ-oscillations by atropine

Cholinergic agonists such as carbachol are used to induce gamma oscillations in hippocampal slices, and atropine blocks these *in vitro* oscillations (Fellous and Sejnowski, 2000; Traub et al., 2000; Whittington et al., 2000; Mann et al., 2005). However we found that atropine increased the amplitude of γ-oscillations at all locations in exploring mice. As somatic inputs are predominantly (or exclusively) inhibitory, the increase in CSD amplitude at the soma indicates that atropine increased inhibition, at least for γ_slow_. The increased inhibition at the soma is likely a result of increased GABA release by the PV-BC's that targets the soma of CA1-PC, mediated by atropine block of the m2 subunit of mAChRs, which are expressed presynaptically on the axon terminals of PV-BC's (Levey et al., 1995; Hájos et al., 1997; Rouse et al., 2000).

In the dendrites, both excitatory and inhibitory inputs are present, so atropine may have altered either inhibition or excitation. We are unable to ascribe changes to specific mechanisms, but the possibilities include blockade of acetylcholine-mediated presynaptic inhibition on glutamatergic and GABAergic synapses (Valentino and Dingledine, 1981; Levey et al., 1995; Qian and Saggau, 1997; Leung and Péloquin, 2010), or reduced feed forward inhibition from CA1 interneurons excited by γ-frequency input from CA3 (Zemankovics et al., 2013). The lack of significant effect on the CFC by atropine suggests that mAChR's are not placed in the crucial position where the slow (θ) and fast (γ) networks interact. The effect of atropine could also be a reflection of changes in the ECtx transmitted to CA1 by changing input strength. However, cholinergic blockade was found to decrease the power of γ-oscillations in medial entorhinal cortex (Newman et al., 2013), making this explanation less likely.

### Implications

Multiple mechanisms have been proposed for generation of gamma oscillations, including interneuron network gamma (ING), pyramidal-interneuron network gamma (PING) and persistent gamma (White et al., 2000; Whittington et al., 2000; Kopell et al., 2010b). Similarly, a number of different models for θ-γ cross frequency coupling have been proposed (Wulff et al., 2009; Kopell et al., 2010a; Buzsáki and Wang, 2012; Buzsáki and Watson, 2012; Tort et al., 2012; Lisman and Jensen, 2013). Given the evidence that multiple γ-oscillations co-exist even within a single brain region, and that they are produced by distinct biophysical and circuit mechanisms, it is possible that each of the different models applies to a specific subset of oscillators, and that there is not a single universally applicable mechanism. Rather, there may be a degree of overlap between these different models acting in tandem to bring about the features of LFPs seen *in vivo*.

### Conflict of interest statement

The authors declare that the research was conducted in the absence of any commercial or financial relationships that could be construed as a potential conflict of interest.

## Supplementary material

Supplementary Figure 1 **Unfiltered raw signal and band-width filtered signals**. The top panel shows a 2-s example of an unfiltered raw signal together with the superimposed θ (4–12 Hz) band-filtered signal. Recording sites 4 and 13 corresponded to *stratum pyramidale* and *stratum lacunosum-moleculare* where the peak of D-AD_pole_ was present. The bottom panels show the γ_slow_(30–45 Hz), γ_mid_(50–90 Hz), and γ_fast_ (90–170 Hz) band-pass filtered segments of the same data as in the top panel. The sections enclosed by differently colored ovals show segments where oscillations occurred in one band but not the others.
