# Supplementary figures and images for: Spatiotemporal characteristics and pharmacological modulation of multiple gamma oscillations in the CA1 region of the hippocampus
Source: Front Neural Circuits. 2015 Jan 12;8:150. doi: 10.3389/fncir.2014.00150 (PMC4290596; doi:10.3389/fncir.2014.00150)

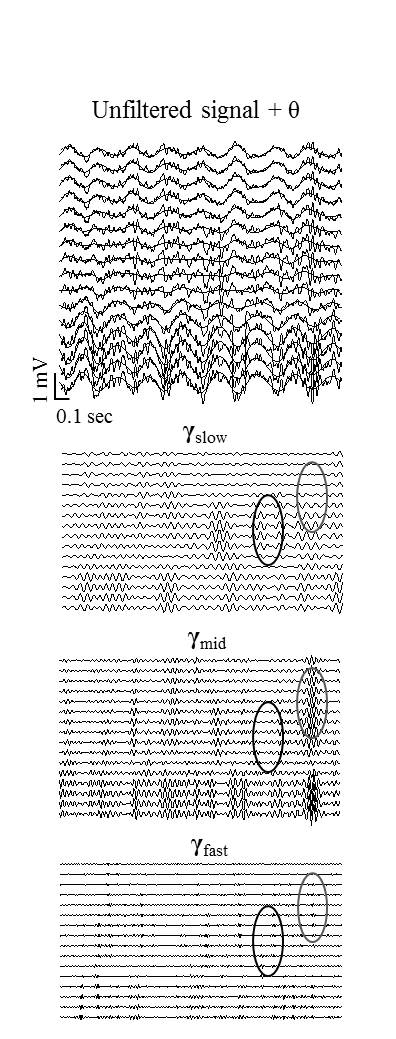

Supplement: Supplementary Figure 1 — Unfiltered raw signal and band-width filtered signals. The top panel shows a 2-s example of an unfiltered raw signal together with the superimposed θ (4–12 Hz) band-filtered signal. Recording sites 4 and 13 corresponded to stratum pyramidale and stratum lacunosum-moleculare where the peak of D-ADpole was present. The bottom panels show the γslow(30–45 Hz), γmid(50–90 Hz), and γfast (90–170 Hz) band-pass filtered segments of the same data as in the top panel. The sections enclosed by differently colored ovals show segments where oscillations occurred in one band but not the others. [file Image1.JPEG]
